# Revisiting the global effect and inhibition of return

**DOI:** 10.1007/s00221-016-4702-9

**Published:** 2016-07-04

**Authors:** Jelmer P. De Vries, Stefan Van der Stigchel, Ignace T. C. Hooge, Frans A. J. Verstraten

**Affiliations:** 1 Division of Experimental Psychology, Helmholtz Institute, Utrecht University, Utrecht, The Netherlands; 2 School of Psychology, The University of Sydney, Sydney, Australia

**Keywords:** Inhibition of return, Global effect, Saccadic landing points, Saccade averaging, Motor attraction, Visual selection

## Abstract

Saccades toward previously cued locations have longer latencies than saccades toward other locations, a phenomenon known as inhibition of return (IOR). Watanabe (Exp Brain Res 138:330–342. doi:10.1007/s002210100709, 2001) combined IOR with the global effect (where saccade landing points fall in between neighboring objects) to investigate whether IOR can also have a spatial component. When one of two neighboring targets was cued, there was a clear bias away from the cued location. In a condition where both targets were cued, it appeared that the global effect magnitude was similar to the condition without any cues. However, as the latencies in the double cue condition were shorter compared to the no cue condition, it is still an open question whether these results are representative for IOR. Considering the double cue condition can provide valuable insight into the interaction of the mechanisms underlying the two phenomena, here, we revisit this condition in an adapted paradigm. Our paradigm does result in longer latencies for the cued locations, and we find that the magnitude of the global effect is reduced significantly. Unexpectedly, this holds even when only including saccades with the same latencies for both conditions. Thus, the increased latencies associated with IOR cannot directly explain the reduction in global effect. The global effect reduction can likely best be seen as either a result of short-term depression of exogenous visual signals or a result of IOR established at the center of gravity of cues.

**Electronic supplementary material:**

The online version of this article (doi:10.1007/s00221-016-4702-9) contains supplementary material, which is available to authorized users.

## Introduction

Due to the limited resolution of the visual periphery and visual crowding, saccadic eye movements are essential to acquire details from visual scenes. While observers typically execute eye movements toward objects of interest at a high rate (3–4 times a second), reaction times can fluctuate depending on events prior to the upcoming movement. For instance, when a peripheral location is cued, initially responses for this location are facilitated after the onset of the cue (up to approximately 200 ms, e.g., Briand et al. 2000). Importantly, facilitation does not just disappear beyond this period, but responses toward the cued location are actually slowed compared to other locations. It is this delay that is referred to as inhibition of return (IOR; Posner et al. 1985).

Despite the fact that IOR was introduced over three decades ago, there is still extensive debate about both the underlying mechanism and its potential functionality. While IOR is typically described as the result of attentional shifts (e.g., Klein 2000), more recently it has been argued to be the result of short-term depression of visual inputs (Satel et al. 2011; Wang et al. 2012b). Regarding the functionality it has been proposed that IOR can be seen as a foraging factor, discouraging the oculomotor system from revisiting previously inspected locations (Klein and MacInnes 1999; Posner and Cohen 1984). Indeed, fixation durations preceding return saccades are longer than for other saccades (Hooge and Frens 2000). Also, when observers had to saccade toward one of two targets, IOR was found to be associated with a spatial bias away from previously visited locations (Boot et al. 2004). However, if IOR would truly serve as a foraging facilitator it should inhibit observers from returning to previously visited locations during actual search tasks. This is difficult to test directly as one cannot simply eliminate IOR in observers. Demonstrating that observers often do return to previously fixated locations during saccadic search tasks and free viewing, it has been argued that IOR is not a foraging factor in search (e.g., Hooge et al. 2005; Smith and Henderson 2011). Arguing that despite the high refixation rates IOR could still be a foraging factor, Bays and Husain (2012) simulated saccadic selection using only instantaneous influences. This revealed that return saccades are more frequent in regular observers than in simulated observers without memory for previously visited locations. Moreover, sequential dependencies between saccades also appear to be consistent with IOR (MacInnes et al. 2014).

Regardless of the functionality, understanding spatial biases associated with IOR can help advance understanding of the oculomotor decision process in general. A study by Watanabe (2001) evaluates how the mechanism behind IOR influences saccade averaging, also known as *the global effect*. The global effect describes the situation where saccade landing points are biased toward the center of closely neighboring elements, rather than landing on a specific one (Coren and Hoenig 1972; Findlay 1982). The global effect is often found to be a time-dependent phenomenon: When the deviation of the saccade landing point is evaluated as a function of saccade latency, the global effect is typically strongest for short latency saccades (e.g., Ottes et al. 1985; Edelman and Keller 1998; Chou et al. 1999; Heeman et al. 2014). In the study by Watanabe, observers were presented with one or two peripheral targets and were required to saccade as quickly as possible toward the single target, or one of the two targets if two were present. In the majority of trials, 600 ms before the onset of the target(s), a single or double cue preceded the target(s) on overlapping locations. When one of the two locations was cued prior to displaying two targets, a spatial bias was obtained: Saccades were biased *away* from the cued target toward the uncued target. This finding is in line with studies demonstrating a spatial bias in the saccade direction (Godijn and Theeuwes 2002; Wang and Theeuwes 2012). When both target locations were cued or both not cued, the global effect appeared unaltered by IOR. This is somewhat surprising given that the global effect magnitude is time-dependent and IOR is associated with slowed responses (i.e., longer response latencies).

Considering the importance of latencies as an indicator for IOR, an issue arises upon inspection of the latencies for the double cue condition in Watanabe (2001). As expected, when a single cue preceded the target, eye movements directed at a previously cued target were delayed compared to when a location adjacent to the target was cued (reflecting IOR). However, when two targets were preceded by a double cue, rather than finding an increase in latencies, saccade latencies were actually *shorter* than when the targets were not preceded by cues. As noted by Watanabe, a likely reason for the lack of delayed responses is the design of the paradigm: While in the no cue condition the targets are preceded by a 2100 ms period of no visual input whatsoever, this same fore-period includes visual cues 600 ms prior to target onset in the double cue condition. The inclusion of the cues provides temporal information on the onset of the targets and can engage attentional systems besides spatial attention (e.g., attentional alerting; Fan et al. 2002). And while such different attentional systems are subtended by independent systems in behavioral tasks they can interact (Callejas et al. 2004). As such, the cue functioning as an alert for the upcoming target may have affected the results of the double cue condition.

Thus, from a strictly phenomenological perspective, IOR was not established in the double cue condition: rather than a delay, facilitation of latencies is found compared to the no cue condition. While it is likely that the mechanisms causing IOR still play a roll, the global effect has been found to depend on latency (Ottes et al. 1985) and has been shown to be modulated by expectation (He and Kowler 1989). Therefore, alerting of target onset in the double cue condition may have affected the magnitude of the global effect even if the mechanisms underlying IOR were in play at the two locations.

### Predictions

While the study of Watanabe (2001) elegantly demonstrates the effect of single cues on saccade averaging, the double cue condition can provide insight on how to best view the relation between the underlying mechanisms of IOR and the global effect. Electrophysiological recordings from the superior colliculus (typically considered the locus of the motor map; e.g., Goldberg and Wurtz 1972) demonstrated that proximal targets were represented individually even when the saccade was executed toward the center of these targets (Edelman and Keller 1998). Consequently, the global effect has been proposed to be the result of competition in the motor map (e.g., Van der Stigchel and Nijboer 2011). If the delay in IOR stems from slowed saccade preparation after the determination of the upcoming landing point, we would expect an unaltered global effect magnitude: The global effect and IOR would each affect sequential parts of saccade preparation that feed into one another. From the perspective of the sequence of events leading up to the saccade, we can say they operate in serial manner. A landing point is determined first, but the saccade that will be executed toward this location is delayed. We will refer to this as the *serial hypothesis*. This hypothesis also encompasses the situation where the inputs that result in saccade averaging would all be equally delayed, simply arriving later in time. Again an unaltered global effect would be expected. Alternatively, it is also possible that the delay in IOR reduces saccade readiness in one stage, while allowing for selection processes to continue longer and resolve the global effect. From the perspective of the sequence of events leading up to saccade execution, the processes can be seen as operating in parallel: While saccade preparation is delayed on one level, saccade selection can progress during the delay. We will refer to this as the *parallel hypothesis*.

Thus even though the question whether inducing IOR by a double cue affects the global effect magnitude remains unanswered, it is certainly an interesting one. The current study is designed to answer this question using a paradigm that minimizes differences between temporal and spatial information in the cues.

## Experiment

In order to evaluate how inducing IOR affects the magnitude of the global effect, we include two conditions: the *Targets Cued condition* where two proximal target locations are cued prior to the onset of two targets. This condition is contrasted to the *Targets Uncued condition*, where the two target locations are not cued. As mentioned above, in Watanabe (2001) the cues provided temporal information on the onset of the targets. Therefore, rather than having no cues at all in the Targets Uncued condition, the same cues as in the Targets Cued condition are presented. The only difference is that the targets do not appear in the same locations as the cues, but in clearly marked locations exactly opposite to the cued locations. In this way, the cues have the same predictive value concerning temporal and spatial target onset characteristics in the Targets Uncued condition as well as in the Targets Cued condition.

## Methods

### Observers

Ten observers (age range 18–25), naive as to the goal of the experiment, participated in the experiment. Observers were recruited through a public website and were paid for their participation. This study was conducted, with written informed consent of each participant, according to the Declaration of Helsinki guidelines.

### Stimuli and apparatus

**Fig. 1:**
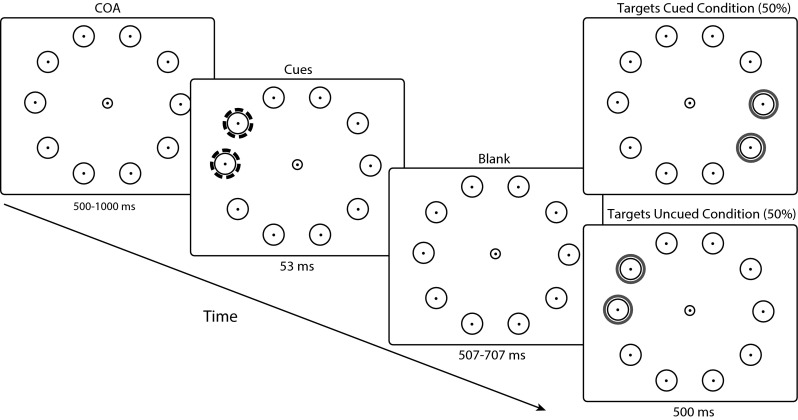
Stimulus chronology. Each trial starts with 10 rings on an imaginary *circle*. Following a COA (cue-onset asynchrony), two red cues (represented by *dotted rings*) are presented at two adjacent locations. The cues are removed after 53 ms, and after a cue-target onset asynchrony of 560–760 ms the two green targets (represented by *solid rings*) appear. On 50 % of the trials these targets appear in the two locations opposite to the previously presented cues (Targets Uncued condition). In the other 50 % of the trials the two green targets appear at the locations of the previously presented cues. The targets remained on screen for 500 ms

Displays consisted of 10 white placeholder disks (1.6° in diameter) with a small black fixation marker in their middle (0.2° diameter). The disks were placed on an imaginary circle with a radius of 9.2°. The disks were present throughout the entire course of the trial (see Fig. 1 top left). We included these disks to have locations to induce IOR at and to facilitate maintenance of IOR at these locations. While there is no evidence that placeholder disks are required to maintain IOR at the cued locations, we did not always find IOR at the desired locations in our pilots initially. Introducing placeholders led to consistent delays at the cued locations and therefore we chose a design where locations are continuously represented by elements on screen. Moreover, the placeholders allowed for a clear indication of where the two locations directly opposite to the cues are.

We should note that while it is not uncommon to use placeholders in establishing IOR (Posner and Cohen 1984; Hunt and Kingstone 2003; Ludwig et al. 2009; Langley et al. 2011), the current paradigm deviates slightly from previous paradigms. In the current case, the cues reduce the number of placeholder locations where the target may appear: Before cuing there are 10 potential target locations, and after the cues this is reduced to 2 (based on the center of the two cues). This information, conveyed by the cues, may create a greater incentive to attend to the cues in general compared to cases where this reduction is not possible. However, cues are rarely completely uninformative in the general sense. For instance, in many studies cues convey temporal information on the onset of upcoming targets as they are presented after an initial cue-onset asynchrony. Also, the potential target locations are located at eccentric positions opposite to each other with respect to the observer’s fixation. Thus, while the chance of attending to the cues is raised, the observer cannot bias attention toward any specific position.

In order to induce IOR we cued placeholders using red rings that were slightly larger than the disks (2.75° diameter; thickness 0.34°). Targets were green rings with the same spatial characteristics.

### Procedure

All trials commenced with a central fixation dot. Upon pressing the space bar the 10 white placeholder disks would appear. Together with the central fixation dot these disks were present throughout the whole trial. After a cue-onset asynchrony (within the range of 500–1000 ms), two adjacent disks (randomly chosen) were cued by two red rings around these disks (see Fig. 1). The cue rings were presented for 53 ms after which they disappeared. After a cue-target onset asynchrony within the range of 560–760 ms two green target rings would appear, either around the locations of the previously cued rings or around the two disks opposite of the cued rings (that is the locations 180° from both the cued locations). Observers were instructed to fixate the central fixation dot until the two green target rings appeared. It was also emphasized to observers that it was important to avoid blinking during this period. While the experimental program did not provide direct feedback on the occurrence of blinks and anticipatory saccades, the experimenter monitoring eye movements on the experimenter PC would notify the observer when frequent mistakes occurred. Trials ended 500 ms after the appearance of the green rings. The observer’s task was to make an eye movement toward one of the two target green rings as fast as possible. All observers performed 448 trials (224 trials mixed for each condition), divided over two blocks that were separated by a small break of 5–10 min.

### Eye movement analysis

 Eye movements were recorded using an SR Research EyeLink II system at a sampling frequency of 500 Hz. The observer’s head was placed in a chinrest so that the eyes were at a distance of 64 cm from the screen. Displays were viewed binocularly, but eye movements were recorded from the left eye only. Eye movement data were collected for off-line analysis. Saccades were detected at a velocity of 20°/s, after which start and endpoint were found by searching for the point (backward and forward, respectively) where the velocity was two standard deviations higher than the velocity during fixation (as in Smeets and Hooge 2003). As our interest is in the saccades from center to peripheral targets, we filter saccades with amplitudes smaller than 0.5°. When a small saccade was removed, the fixations before and after this saccade were added together. Moreover, fixation durations shorter than 25 ms were discarded from further analysis.

The period from the onset of the placeholders to target onset lasted between 1050 and 1750 ms. While observers were instructed to maintain fixation during this period, some anticipatory saccades and blinks are inevitable over the course of the experiment. Therefore, the following criteria were applied (percentages of trials where this error occurred is shown in parentheses behind them): Trials on which movements larger than 1.5° (this also includes blinks) were detected prior to target onset were discarded (occurs on 4.8 % of trials). Trials on which the eye was not fixating within 1.5° of the fixation dot (3.9 %). Saccades with latencies shorter or longer than 90 and 500 ms, respectively, were excluded (2.3 %). To ensure saccades were properly directed toward the target, we excluded trials on which the amplitude of the saccade was under 70 % or over 130 % of the target eccentricity (8.5 %). For the same reason, saccadic landing points deviating more than 36° from one of the two targets (with respect to the central fixation dot) were also excluded (2.0 %). Combining these criteria led to an exclusion of 14.7 % percent of the trials. Please note, as several trials will have a combination of errors, this number is less than the sum of the above percentages.

## Results

### IOR manipulation

**Fig. 2:**
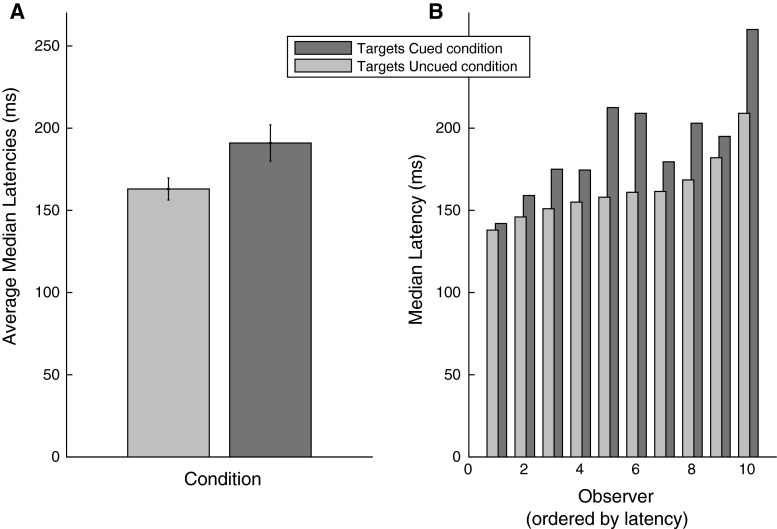
Latencies for saccades per condition. The *light gray bar* represents the latencies from the Targets Uncued condition. The *dark gray bar* represents the latencies from the Targets Cued condition. *Error bars* represent standard error of the mean

In order to assess whether the IOR phenomenon was properly established at the cued locations, we compare the latencies of saccades directed at targets at the previously cued locations, versus saccades directed at targets that were not cued. In Fig. 2a median latencies of all the observers are averaged for the conditions, separately, and in Fig. 2b the median latencies of individual observers are shown for both conditions. Latencies for targets on cued locations were longer than the latencies for saccades toward not cued locations (average 28 ms, *t*(9) = 4.9343, *p* < 0.001, Cohen’s *d* = 2.168). Moreover, while latency differences vary, for each individual observer we found longer latencies in the Targets Cued condition than in the Targets Uncued condition (Fig. 2b). Thus, we can properly refer to this condition as the Targets Cued condition.

### Global effect

**Fig. 3:**
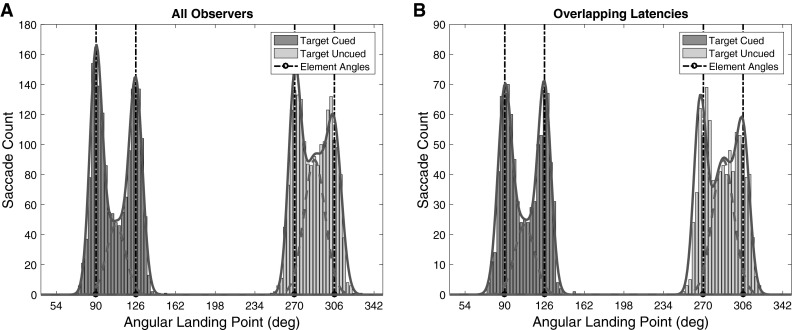
Landing positions (in degrees) of binned saccades (bin width equals 3°). **a** Contains all saccades collapsed over all observers for both conditions. Landing positions from the Targets Cued condition are colored *dark gray*; those from the Targets Uncued condition are colored *light gray*. *Vertical bars* represent the target positions. These are oriented such that they were 90° and 126° in the Targets Cued condition and 270° and 306° in the Targets Uncued condition. Note this means that the two cues always stood at 90° and 126°, respectively. In the *blue line*, a fit of equation 1 (the summation of the three Gaussians) can be seen. Alternatively, the *red dotted line* represents only the global Gaussian component of this function. **b** Landing positions for saccades with overlapping latencies collapsed over all observers for conditions. Again the blue line represents a fit of equation 1, while the *red dotted line* represents only the global Gaussian component. Note that the fits in the current figure are purely for illustrative purposes. Statistical analysis was purely based on fits of individual observers that can be found in supplemental Figures S1 and S2 (color figure online)

To evaluate the magnitude of the global effect, we analyzed the saccadic landing points. In our experiment, the two targets stood at an angular separation of 36° (as seen from the central fixation point). A typical global effect would consist of many eye movements landing between the two target locations. Therefore, we determined the angular direction of each saccade based on its landing position. To evaluate performance over trials, all angles were rotated as if the two cues were presented at 90° and 126°. Through this rotation the target locations stand at 90° and 126° for the Targets Cued condition and at 270° and 306° for the Targets Uncued condition. Subsequently, the rotated angles were binned in bins spanning an angular width of 4.5° (for individual observers) that were centered on the presented elements. In Fig. 3a we present the number of eye movements falling inside each bin both for individual observers and all trials collapsed over observers. Comparing the two conditions, the global effect appears stronger in the Targets Uncued than in the Targets Cued condition.

To establish whether this difference can be generalized beyond our sample, we estimated the difference in global effect magnitude between the two conditions for each observer, individually. Previous studies have established the magnitude of the global effect in various ways. One solution is to classify landing points using predefined areas (Edelman and Keller 1998; Van der Stigchel et al. 2011). When a single target condition is available, landing points toward double targets can be compared to the landing point distributions of the single target (e.g., Chou et al. 1999). As a single target condition is not available in our experiment, we use a fitting procedure to distinguish global effect saccades from saccades properly directed toward the individual elements. Naturally even eye movements directed at a single target will not always fall exactly on the center of the target, rather landing points will be distributed around its center. Eye movements deviating toward the neighboring target ring can, therefore, be both the result of a typical deviation around the center of the targets themselves, as well as the result of the presence of the neighboring target (the global effect). Therefore, we propose that an appropriate manner to establish the magnitude of the global effect is to fit the landing point angles using the sum of three Gaussians. Two Gaussians on the ring positions and one centered in the middle of the two rings as by the following equation

1 $$\begin{aligned} {\text{saccade}}_{\text{count}} & = e^{{\frac{{ - \left( {x - \mu_{\text{center}} } \right)^{2} }}{{ {2\left( {\sigma_{\text{center}} } \right)^{2}} }}}} {a_{\text{center}} } \\ & \quad + e^{{\frac{{ - \left( {x - \mu_{\text{left}} } \right)^{2} }}{{ {2\left( {\sigma_{\text{left}} } \right)^{2}} }}}} {a_{\text{left}} } + e^{{\frac{{ - \left( {x - \mu_{\text{right}} } \right)^{2} }}{{ {2\left( {\sigma_{\text{right}} } \right)^{2}} }}}} {a_{\text{right}} } \\ \end{aligned}$$

Here, *μ*_left_, *μ*_right_, and *μ*_center_ refer to the positions of the left target, right target, and the position in between, respectively. In a similar manner *a*_left_, *a*_center_, and *a*_right_ refer to the amplitudes and *σ*_left_, *σ*_center_, and *σ*_right_ to the deviations. In order to reduce the number of free parameters, we set some positions based on the data. The amplitude and position of the left Gaussian are set to the bin with the greatest number of eye movements directed toward the left element. Note, however, allowing the mean position of the left Gaussian to move toward a position between the two targets would allow for absorbing a stronger global effect in one of the conditions. To prevent this we have restricted the mean of the left Gaussian from moving inward beyond the center of the left element. While restricting the range of the outer Gaussians may slightly exaggerate the global effect magnitude, importantly, this holds equally for both conditions. Subsequently, the same holds for the right Gaussian, where the amplitude and mean are set to the bin with the greatest number of eye movements including the central bin on the right element and those to its right. Moreover, *μ*_center_ is set to the position exactly in between the two elements. This leaves four free parameters *σ*_left_, *σ*_center_, *σ*_right_, and *a*_center_. We fit these using a least squares estimation procedure (*nlinfit* function of the statistics toolbox in MATLAB; The MathWorks, Natick, MA). The result of the fitting procedure is plotted in Fig. 3a on top of the histogram (fits for individual observers can be found in supplemental Figure 1). The blue line represents the result of the above equation fitted to the observer’s data, while the red dotted line represents only the central Gaussian component. We take the global effect magnitude to be the proportion of the area under the red dotted curve divided by the area under the blue curve (representing the summation of the three Gaussians). In order to evaluate whether the decrease in global effect magnitude can be generalized over our observers, a *t* test was performed. Comparing the global effect magnitude (area under the red curve divided by the area under the blue curve) shows it was significantly greater in the Targets Uncued condition than in the Targets Cued condition (*t*(9) = 3.7861, *p* < 0.005, Cohen’s *d* = 1.211).

### Latency-dependent analysis

**Fig. 4:**
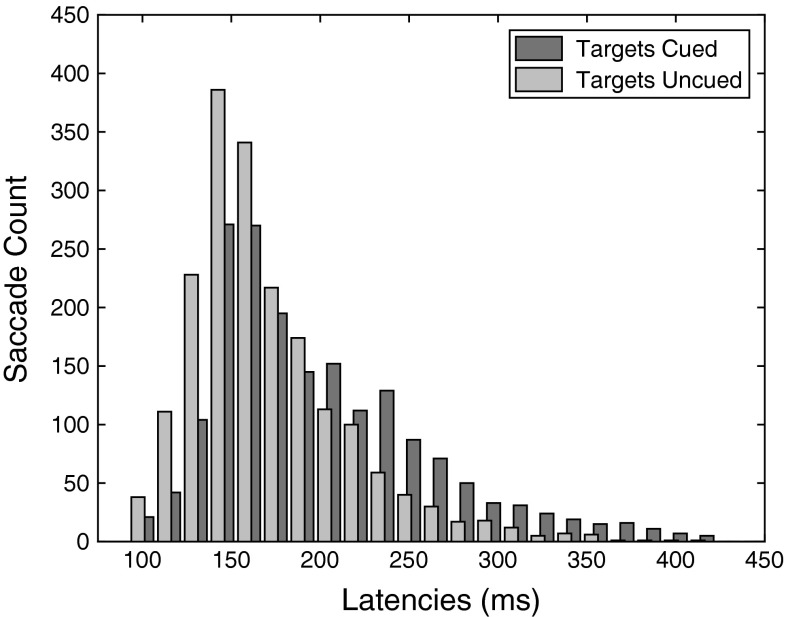
Latency histogram for both the Targets Cued condition and the Targets Uncued condition collapsed over observers. Despite longer latencies for the Targets Cued condition, there is considerable overlap in the distributions

Based on evidence that the global effect is typically less strong for longer latency saccades (Findlay 1982; Ottes et al. 1985), we hypothesized that any decrease in global effect magnitude in the Targets Cued condition would be directly related to the prolonged latencies in this condition (compared to the Targets Uncued condition). To evaluate whether the longer latencies can explain the decrease we performed the same comparison as above, but now only including saccades with latencies that are equal in both conditions (despite longer median latencies in the Targets Cued condition than in the Targets Uncued condition the latency distributions still overlap considerably, see Fig. 4). If longer latencies are indeed the sole reason for the decreased global effect magnitude, a similar global effect magnitude for the overlapping parts of the latency distributions is to be expected.

Saccades with the same latencies over the two conditions were identified (for each observer individually), and landing positions for these saccades were determined as above. Naturally, the number of saccades with a particular latency will not always be the same over both conditions. Identification of saccades with matching latencies was performed by chronologically traversing trials from the Targets Uncued condition and for each of them finding a trial in the Targets Cued condition with the same initial saccade latency. Trials for which no matches were found were dropped from the analysis. This approach resulted in an average of 47.3 % overlapping latency saccades for our observers. The overlapping data are shown in Fig. 3b in a similar manner as in Fig. 3a (fits for individual observers can be found in Supplemental Figure 2, because of less eye movements we now use bin widths of 6°). Despite using only saccades with the same latencies, there still appears a considerable difference in global effect magnitude between the conditions. Performing the fitting procedure as described above for each observer and comparing the global effect magnitude indeed demonstrate a significant difference between the two conditions (*t*(9) = 3.1915, *p* < 0.05, Cohen’s *d* = 1.0626). Hence, the latency difference by itself cannot explain the difference in global effect magnitude.

### Latency at landing point analyses

With the prolonged latencies in the Targets Cued condition, we find a reduced global effect magnitude. As such our data appear to be in line with the parallel hypothesis outlined in the introduction predicting a reduction in global effect magnitude. However, the hypothesis stated that the delay would lead to extra time for proper selection of either of the two targets. Considering we find a reduced global effect even when analyzing saccades with the same latencies, the finding does deviate from the parallel hypothesis. An alternative explanation can be deduced from two previous studies investigating the use of multiple cues in IOR (Christie et al. 2013; Klein et al. 2005). When presenting multiple cues, IOR was influenced more by the center of gravity of the cues than the individual cue locations. In our paradigm, this could mean that IOR was established at the central position, or at least IOR could be stronger at the central position than at the actually cued positions. This makes sense as naturally IOR extends over a certain range from its epicenter; see, for instance, Hooge and Frens (2000). In this case, the diminished global effect magnitude could be the result of a spatial bias away from the location where IOR was established.

**Fig. 5:**
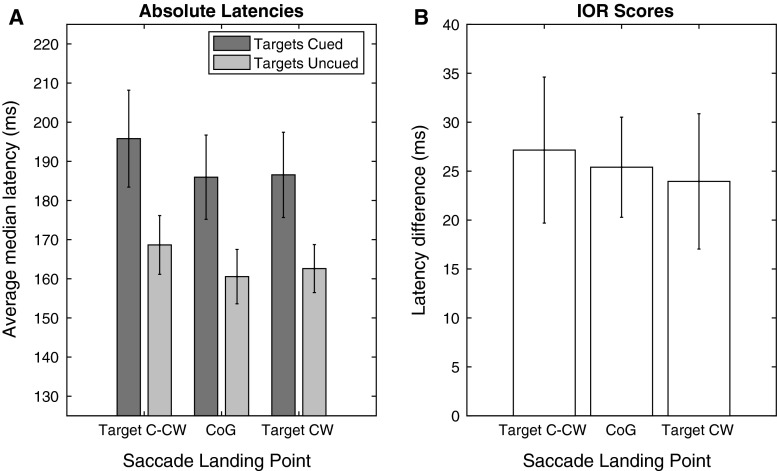
Latencies based on saccade direction. **a** Latencies for the Targets Cued condition (in *dark gray*) and the Targets Uncued condition (in *light gray*), separately. **b** The strength of IOR as calculated by taking the absolute difference between the Targets Cued condition and Targets Uncued condition. *Error bars* represent standard errors of the mean

To explore whether IOR was stronger at the center and the center-of-gravity account is the best explanation for the current results, we evaluated the strength of IOR (i.e., the difference between latencies in the Targets Cued and Targets Uncued condition) for saccades directed at the individual elements, as well as those directed toward the center. To this end, saccades were divided over three bins. Saccades with an angular landing position at a separation of <12 angular degrees from the center were classified as directed toward the center of gravity (the inner 24° of the 36° separation). Landing positions counterclockwise from this range were classified as directed toward the counterclockwise target. Saccades with landing positions clockwise from this range were classified as directed at the clockwise target. The average of the median latencies is plotted in Fig. 5a. If stronger IOR at the center of gravity explains the current result, we would expect to see a larger latency difference between conditions on the central bin than on the bins corresponding to the clockwise and counterclockwise targets. In Fig. 5b IOR score is calculated by subtracting the latencies of the Targets Uncued condition from the latencies of the Targets Cued condition. It appears we do not find stronger IOR at the center of gravity. To minimize the possibility of missing potential differences between the different IOR scores, we ran two separate *t* tests comparing IOR scores from the bins representing the elements with the bin representing the middle position. We did not find a significant difference comparing IOR at the left position to the middle (*t*(9) = 0.2368, *p* = 0.8181) nor at the right position to the middle (*t*(9) = 0. 2855, *p* = 0.7818).

Given the lack of stronger IOR at the center of the cues, we cannot conclude that the center-of-gravity account was the driving factor behind the reduced global effect magnitude in the Targets Cued condition. Conversely, we should emphasize that we also cannot completely exclude the possibility that the center-of-gravity account did not play a role here either. It is, for instance, possible that IOR at the center of the two cues biased eye movements away from the center. In such a case, eye movements that do land in the center may be those that were least affected by IOR. This would lower the IOR score for the central bin. However, this may also affect the latency of the eye movements that were biased away from the center, as they should also be relieved from IOR. In essence it is likely that with the alteration of the spatial distribution of saccades IOR scores may be biased. While finding a clear center-of-gravity effect would have been a strong indication that the reduction in global effect magnitude was the result of IOR located in between the cues, a lack of the center-of-gravity effect does not necessarily exclude the possibility completely.

## Discussion

Watanabe (2001) evaluated the behavioral interaction between the global effect and IOR. While the study provides valuable insights into this interaction in the single cue condition, the typical delay associated with IOR was lacking in the double cue condition. This was likely due to the predictive value of the cues in the double cue condition that were lacking in the no cue condition (as indicated by Watanabe himself). As the global effect is typically found to be dependent on latency (e.g., Ottes et al. 1985; Edelman and Keller 1998) and expectation plays a role in saccade averaging (e.g., He and Kowler 1989), this may have obscured changes to the global effect magnitude. The global effect is often reasoned to be the result of averaging of the saccade vectors elicited by two proximal targets when there is not enough time for top-down selection to play a role (e.g., Van der Stigchel and Nijboer 2011). In line with this notion, activity for proximal targets in the superior colliculus (often considered the motor map for saccades) resembles the individual targets for express saccades that target the center (Edelman and Keller 1998). Using an adapted paradigm, we ensured latencies were increased for all observers in the double cue condition. Interestingly, with this we also find a decrease in global effect magnitude when IOR is established at both target locations compared to when it is not established at the target locations.

As inducing IOR appears to decrease the global effect magnitude, we can exclude any explanation that does not predict IOR to decrease the global effect magnitude. For instance, if IOR would delay saccade execution after the resolution of the saccadic landing point, we should not have found a decrease in global effect magnitude. Similarly, in the current case IOR cannot be seen as a pure delay in inputs from higher areas of visual processing as this would not lead to a resolution of the global effect. Returning to the hypotheses presented in the introduction this means we can reject the serial hypothesis. Rather it appears that the global effect and IOR interact in line with the parallel hypothesis proposed in the introduction. However, in the introduction we suggested that such an interaction could be the direct result of the delay in latencies in IOR: As the global effect is typically strongest for the short latency saccades, the longer latencies in the Targets Cued condition could allow for further resolution of saccade landing points. Nevertheless, despite finding a diminished global effect, it does not appear to be the result of delayed latencies: Even for saccades with the same latencies, there was a significant decrease in global effect magnitude in the Targets Cued compared to the Targets Uncued condition.

Despite previous findings demonstrating that the global effect is often stronger for shorter than for longer latencies, we do not find the expected effect of latency. While indeed the global effect has been reported to be stronger in several studies, it is important to realize that many studies finding a time-dependent global effect (decreasing magnitude for longer latencies) use a less conspicuous target and more conspicuous (also known as *salient*) distractor. However, in a previous study employing two identical targets and no explicit instruction to prefer one over the other, the global effect was not dependent on the latency of the saccade (Van der Stigchel et al. 2012). When two different targets were used, a difference was only found when there was an explicit instruction to favor one element over another (Heeman et al. 2014). Potentially, when visual target selection is not biased toward one target over the other, more time for visual target selection does not decrease the central bias.

Elaborating on the findings of Watanabe (2001), Wang et al. (2012a) showed that the bias away from cued location does not only occur for long cue-target onset asynchronies (CTOAs; those typically resulting in IOR), but also with shorter CTOAs. As shorter latencies are typically associated with the notion that an element is an attractive saccade target, the bias away from the target is counterintuitive. As this landing point deviation seems to be related to cuing rather than IOR specifically, the question arises whether the current result stems from IOR or cuing in general. A strong indication that there is indeed a relation between IOR and the reduction in global effect magnitude stems from Watanabe (2001). In Watanabe’s study, where the typical delay associated with IOR was not observed, a decrease in global effect magnitude was also not observed for double cues. Conversely, in our experiment we do find delays in latencies typically associated with IOR as well as a reduced global effect. The fact that with the cuing and shorter latencies (as may be expected with shorter CTOAs) the global effect magnitude does not decrease indicates that it is likely that the current results are linked to IOR rather than cuing in general.

This leaves us with the question why establishing IOR would lead to a diminished global effect even when there is no delay in latencies. A plausible explanation can be deduced from a recent proposal by Satel et al. (2011) that advocates IOR to be a side effect of short-term depression of visual inputs. This proposal is in line with data that demonstrate that inputs to the superficial layers of the superior colliculus are diminished, but the superior colliculus is not the site of inhibition itself (Dorris et al. 2002). These direct inputs to the superficial layers are typically thought to represent the exogenous inputs of saccadic selection, unaffected by the observer’s intentions. Considering that the global effect is often reasoned to be the result of bottom-up responses, a reduction in this exogenous input would diminish the bottom-up attraction of the two targets. In this case, the weight of slower endogenous inputs will increase as the landing point will not yet be established when they arrive. Thus, short-term visual depression may diminish the bottom-up attraction of the two elements and allow for more effective top-down selection of the target.

However, would top-down inputs not also be suppressed? As physiological recordings show that the depressed response in early visual processing is propagated throughout the rest of the brain (Fecteau and Munoz 2005) this is likely. Nevertheless, the observer’s intentions do not change and therefore top-down selection will be continuous, in contrast to the transient bottom-up response. The depressed top-down selection may therefore be responsible for the slowed response, but will still be a dominating factor in saccadic selection as the intention of the observer is not transient. Thus, the resulting interaction between bottom-up and top-down processes could explain why observers are better at directing their eye movements to the target individually when IOR is established at the target locations.

A second alternative is that rather than depressed exogenous signals, the double cue results in IOR at the point directly in between the cues. Two recent studies have demonstrated that when multiple cues are used, IOR is stronger toward the center of gravity of the cues compared to the individual locations (Christie et al. 2013; Klein et al. 2005). As IOR at the center of the two cues could introduce a spatial bias counter to the typical global effect bias, this could explain the decreased global effect magnitude. If that were the case, we would expect the strength of IOR (difference between the latencies of the two conditions) to be strongest for saccades directed toward the center of gravity of the cues. In a follow-up analysis of the latency difference, we did not find such a pattern. Although this may seem in contrast to the findings of Klein and colleagues, it is still possible that IOR was located at the center of the cues. For instance, it is also possible that IOR was established in between the two cues, but the small spacing from center to the flanking cues is not sufficient to detect a drop off with distance in IOR. Alternatively, the alteration of the spatial distribution may have obscured the center-of-gravity effect. For instance, it is possible that the few saccades that do land in the center are a selective sample: If IOR at the center of gravity is the cause of the reduction of the global effect magnitude, potentially only faster saccades were able to break through the inhibition. However, we can only speculate to what extent IOR at the center of gravity would affect the spatial distribution and the latencies of the saccades associated with them. Importantly, while we do not find direct evidence for a center-of-gravity effect in the current data we cannot completely exclude the possibility that it played a role either.

In conclusion, demonstrating that the global effect magnitude is decreased as a result of inducing IOR, the current paper provides an important addition to previous findings on saccade averaging and IOR. The reduction can likely best be seen as either reduced bottom-up activity as a result of short-term visual depression (Satel et al. 2011) or a result of IOR established at the center of gravity of cues (Christie et al. 2013).

## Electronic supplementary material

Below is the link to the electronic supplementary material.

Figure S1 Fits for individual observers for saccades from both conditions. For each observer the fit of equation 1 in blue (representing the sum of the three Gaussians). The global Gaussian component is represented by the red dotted line. The Target Cued condition is indicated in dark grey, while the Target Uncued condition bins are light grey (EPS 348 kb)

Figure S2 Fits for individual observers for saccades with overlapping latencies. For each observer the fit of equation 1 in blue (representing the sum of the three Gaussians). The global Gaussian component is represented by the red dotted line. The Target Cued condition is indicated in dark grey, while the Target Uncued condition bins are light grey (EPS 250 kb)
